# Integrated top-down and bottom-up mass spectrometry enables precise characterization of proteoforms and their post-translational modifications within the protein corona

**DOI:** 10.21203/rs.3.rs-7593385/v1

**Published:** 2025-09-16

**Authors:** Morteza Mahmoudi, Seyed Sadeghi, Kun Li, Yifan Yue, Reyhane Tabatabaeian Nimavard, Shaun Grumelot, Amir Saei, Hojatollah Vali, Xiaowen Liu, Liangliang Sun, Fei Fang

**Affiliations:** Michigan State University; Michigan State University; Tulane University; Michigan State University; Michigan State University; Michigan State University; Department of Microbiology, Tumor and Cell Biology, Karolinska Institutet, Stockholm, Sweden.; McGill University; Tulane University; Michigan State University; Michigan State University

## Abstract

Precise characterization of proteins and proteoforms within the protein corona is essential for developing safer and more effective nanomedicines for diagnostic and therapeutic applications. Although the protein corona phenomenon has been recognized in nanomedicine for nearly two decades, the application of top-down proteomics to analyze proteoforms within this context has only recently gained traction. In this study, we advance proteoform-level analysis of the protein corona by integrating mass spectrometry (MS)-based top-down proteomics (TDP) and bottom-up proteomics (BUP). TDP analysis of protein corona of polystyrene nanoparticles (PSNPs) identified 3,505 proteoforms of 344 genes in human plasma samples, representing nearly 4-fold improvement in the number of proteoform and gene identifications (IDs) from protein corona of PSNPs and the largest proteoform dataset of protein corona reported so far. BUP analysis of the protein coronas identified 4,570 protein groups, 45,790 peptides, and 23,632 peptides containing modifications in the human plasma samples, representing one of the most comprehensive plasma proteome datasets from BUP to date and over 150% increase in protein IDs compared to previous PSNP–based corona studies. The combination of such large TDP and BUP datasets improves the characterization quality of nearly 35% of identified proteoforms containing mass shifts, producing a more precise proteoform landscape of protein corona. This BUP and TDP combination approach exceeds the capabilities of individual techniques for proteoform characterization in protein corona, and will eventually enhance our understanding of the protein corona and offer valuable insights into nanoparticle–biosystem interactions, as well as advancing proteoform-level biomarker discovery.

Nanoparticles (NPs) have become integral to various applications in nanomedicine, serving roles in targeted drug delivery, imaging, and diagnostics.^1–8^ When NPs are introduced into biological systems/fluids, they rapidly acquire layer(s) of biomolecules from the surrounding environment, predominantly proteins, forming what is known as the protein corona.^9^ This dynamic corona defines how the NP interacts with cells and tissues, influencing its biological identity, pharmacokinetics, and overall efficacy.^10^ Consequently, understanding the composition and structure of the protein corona is crucial for the rational design of nanomedicine strategies and for predicting biological responses.^11^

Mass spectrometry (MS)-based proteomics has long been the primary technique for profiling the proteins within the corona. Until 2024, the dominant approach relied on bottom-up proteomics (BUP) ^11–13^, which involves enzymatic digestion of proteins into peptides, followed by separation and MS analysis. While BUP provides high coverage of peptide fragments and enhances the localization of post-translational modifications (PTMs), it falls short in accurately identifying proteoforms—the specific molecular variants of proteins—including their combinatorial PTMs. This limitation stems from the enzymatic digestion step, peptide loss during digestion, and the inherent peptide-to-protein inference problem, which complicates the reconstruction of full proteoform identities.^14,15^ Proteoforms arising from sequence variation and PTMs can exhibit distinct biological functions ^16–19^ and play pivotal roles in disease progression ^20–23^. For example, PTMs on human serum albumin (HSA) influence its binding interactions with NPs, impacting corona thickness and NP–cell interactions.^24^ As such, precise characterization of proteoforms within the corona is critical for understanding NP–cell dynamics and advancing proteoform-based biomarker discovery.

Very recently, we developed an efficient and reproducible top-down proteomics (TDP) platform for analyzing the proteoforms directly, by measuring intact proteins without enzymatic digestion.^25^ This approach preserves full proteoform information and has been further refined through the incorporation of diverse separation techniques and varying NPs, significantly improving proteoform identification.^26–28^ Despite these advancements, TDP still faces challenges, notably limited backbone cleavage coverage of proteoforms, which hinders the precise localization of PTMs.^29^

Combining BUP and TDP MS strategies leverages their complementary strengths for comprehensive PTM characterization, the feature that cannot be defined by either of techniques (Fig 1). TDP provides insights into proteoform diversity and PTM patterns, while BUP offers high backbone cleavage coverage of peptides, facilitating accurate PTM localization and validation. In this study, we explore the synergistic potential of this integrated approach by analyzing protein coronas formed on polystyrene NPs (PSNPs). Proteoform-level details were obtained through TDP, while peptide-level analysis was performed via BUP, with data integration facilitated by the PTM-TBA (top-down and bottom-up MS and annotations) software pipeline^30^. Our findings demonstrate that this combined strategy yields unprecedented accurate localization of modifications on specific proteoforms in protein corona; such information is unattainable by either approach alone and, therefore, the combination strategy may open new avenues for proteoform-focused biomarker discovery and understanding nanoparticle–biosystem interactions.

## Results

**Figure 2:**
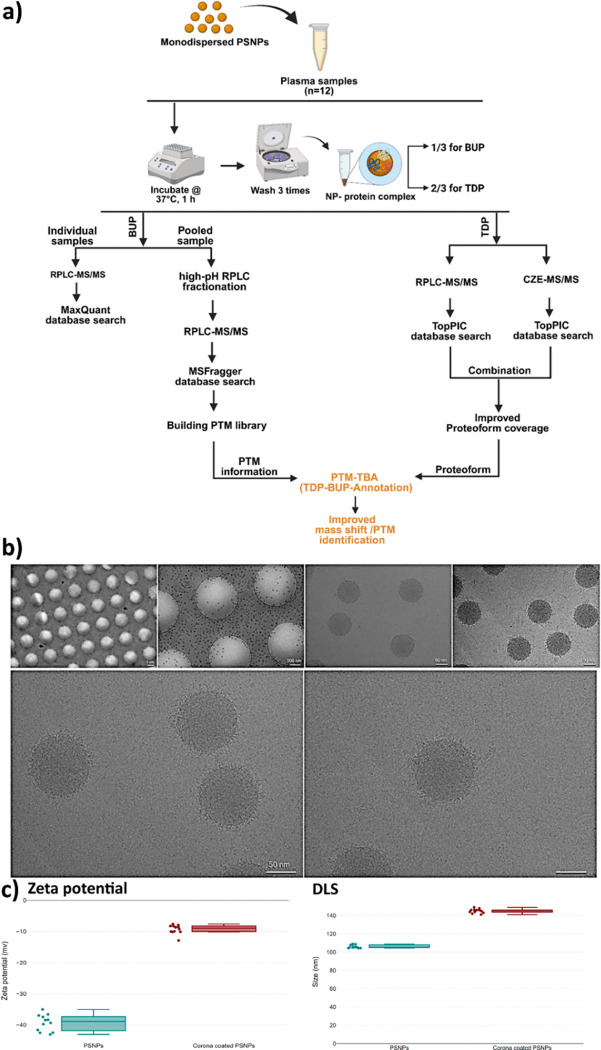
Characteristics of protein corona coated nanoparticles. **(a)** displays the overall workflow integrating top-down proteomics (TDP) and bottom-up proteomics (BUP). Pure corona-coated PSNPs were analyzed using both approaches to enable comprehensive identification of proteoforms and proteins. Subsequently, the integrated PTM-TBA pipeline was employed for detailed proteoform characterization and precise localization of post-translational modifications (PTMs). **(b)** The cryo-TEM images of protein corona coated PSNPs at different magnifications. The generated movie of the 3D reconstruction of the protein corona using electron tomography is available in **Supplementary Movie 1**. **c)** Zeta potential and dynamic light scattering (DLS) analysis of PSNPs before and after formation of protein corona.

The protein corona formed on the surface of polystyrene nanoparticles (PSNPs) was fully characterized using cryo-transmission electron microscopy (cryo-TEM), dynamic light scattering (DLS), zeta potential, and analyzed through both BUP and TDP MS (Fig. 2a–c). All the generated BUP and TDP data are listed in **Supporting Data 1**. Ensuring that the corona is free from significant aggregation or protein contamination is essential for accurate characterization of the protein-nanoparticle interactions.^31^ Cryo-TEM images demonstrated highly monodispersed, protein corona-coated PSNPs, confirming the successful formation of a uniform and pure corona layer (Fig. 2b). DLS and zeta potential measurements conducted before and after corona formation showed consistent results indicating successful coating: the nanoparticle size increased, reflecting the presence of the protein corona, while the surface charge became less negative post-coating (Fig. 2c). These observations are in full agreement with reported literature findings, supporting the reproducibility and reliability of the corona formation process^32–39^.

**Figure 3:**
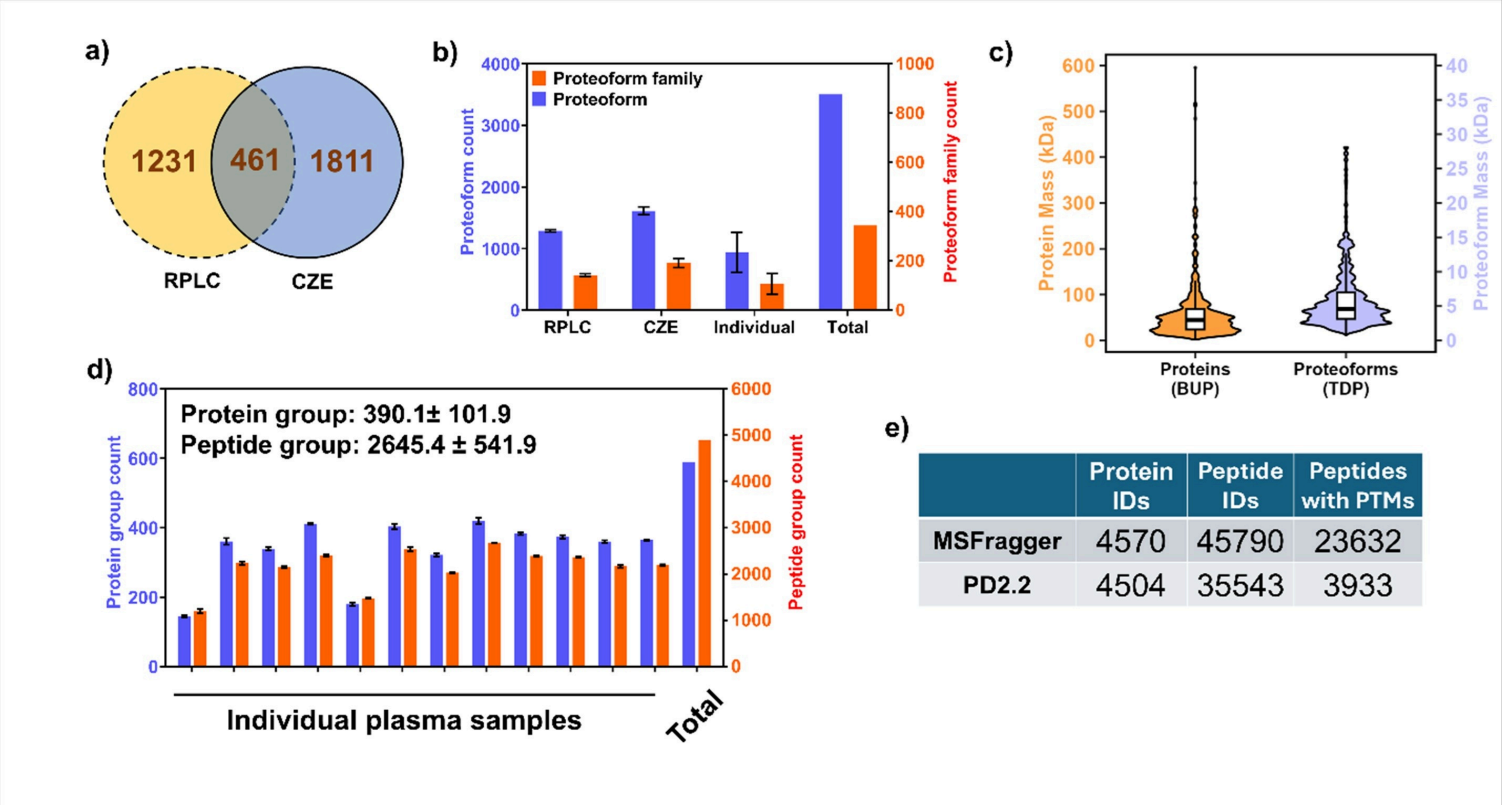
Proteoform and protein identifications (IDs)across different analytical workflows and sample groups. (a) Venn diagram illustrating the number of unique and shared proteoforms identified by RPLC–MS/MS, CZE–MS/MS. (b) Bar plots showing the numbers of proteoforms and proteoform families detected from the human plasma samples using RPLC and CZE separation techniques. The error bars for RPLC and CZE show the standard deviations across technical triplicate runs; the error bars for Individual represent the standard deviations across 12 different samples. (c) Combined violin and box plots depicting the mass distributions of proteoforms (from TDP) and proteins (from BUP). (d) Summary of bottom-up proteomics results, including the numbers of protein groups and peptide groups identified per sample. The error bars represent the standard deviations across triplicate LC-MS analysis. (e) The number of proteins, peptides, and peptides with PTMs identified by 2D high-pH RPLC-low pH RPLC-MS/MS from the pooled protein corona peptide sample using two different database search approaches (MSFragger open-search and Proteome Discoverer 2.2 (PD2.2) SEQUEST HT).

To achieve a more robust and comprehensive understanding of how integrating BUP and TDP enhances the accuracy and reliability of proteoform characterization for protein corona, we need to have a large protein corona proteoform dataset. To produce this proteoform dataset, we analyzed a spectrum of protein coronas from various human plasma samples, for example, three samples from healthy individuals, five samples from patients with grade I breast cancer, and four samples from patients with grade II breast cancer. The diversified proteome profiles of human plasma samples from various individuals and health conditions help improve the number of proteoform identifications from protein coronas^12^. The proteoform profiles of protein coronas from diverse individuals could also reflect the biological variability associated with personalized and disease-specific factors^40^. We also employed two different measurement approaches, capillary zone electrophoresis-tandem mass spectrometry (CZE-MS/MS) and reversed-phase liquid chromatography (RPLC)-MS/MS (Fig. 2a), to boost the number of proteoform identifications from protein coronas, because these two approaches have been well documented for complementary peptide/proteoform identification from complex proteomes^25,41–47^.CZE-MS/MS identified 2,272 proteoforms and 283 proteoform families—approximately 34% more proteoforms (2,272 vs. 1,692) and 50% more families (283 vs. 189) than RPLC-MS/MS. The relatively low overlap of proteoforms between the two methods highlights their strong complementarity in enhancing the depth of corona proteoform analysis, Fig. 3a. By collectively analyzing protein coronas from 12 human plasma samples, we identified a total of 3,503 proteoforms corresponding to 344 genes, Fig. 3b. Interestingly, the protein corona proteoform profiles of the three types of human plasma samples (healthy control, grade I breast cancer, and grade II breast cancer) are substantially different, evidenced by the low proteoform overlaps among the three sample types, **Fig. S1.**

While TDP enabled the detection of intact proteoforms in the protein corona, it alone was insufficient for comprehensive PTM characterization, primarily due to incomplete backbone cleavages that limited precise PTM localization. To address this, we integrated TDP with two BUP experiments, Fig. 2a. In the first BUP experiments, one-third of each corona sample was digested and analyzed by RPLC-MS/MS in triplicate. In this experiment, we identified an average of 390 protein groups and 2,645 peptide groups per sample (Fig. 3d), totaling 588 unique proteins and 4,899 unique peptides across all samples (Fig. 3d). The protein mass from BUP is up to 600 kDa and the TDP data only covers proteoforms smaller than 30 kDa, Fig. 3c, which represents another technical challenge of TDP regarding large proteoform identification. In the second BUP experiment, we aim to create a much larger peptide dataset to cover more PTM information for better interpretation of TDP data. We pooled the leftover peptide materials from all 12 human plasma samples to produce a more complex peptide mixture and employed high-pH RPLC fractionation followed by nanoflow RPLC-MS/MS to analyze the sample. To maximize the PTM information, we utilized an open-search approach with MSFragger^48^. We identified 4570 protein groups, 45790 peptides, and 23632 peptides containing modifications, *e.g.*, glycosylation, phosphorylation, acetylation, oxidation, and deamidation. The number of protein IDs in this study represents one of the largest human plasma proteome datasets in one study and is more than 150% higher than that from previous polystyrene NP-based protein corona studies^49,50^. The large number of peptides with PTMs allows us to establish a PTM library for the PSNP-based protein corona. We also performed another database search using Proteome Discoverer (PD2.2, SEQUEST HT) and identified 4504 protein groups, 35543 peptides, and 3933 peptides with PTMs. The number of peptides with PTMs is much smaller compared to MSFragger because we only specified several specific PTMs (i.e., oxidation, acetylation, methylation, succinylation, and phosphorylation) in the PD search. We then integrated the BUP and TDP datasets using PTM-TBA to enhance the characterization quality of proteoforms, particularly in terms of annotation and localization of PTMs. We mainly used the MSFragger BUP data for this purpose.

**Figure 4:**
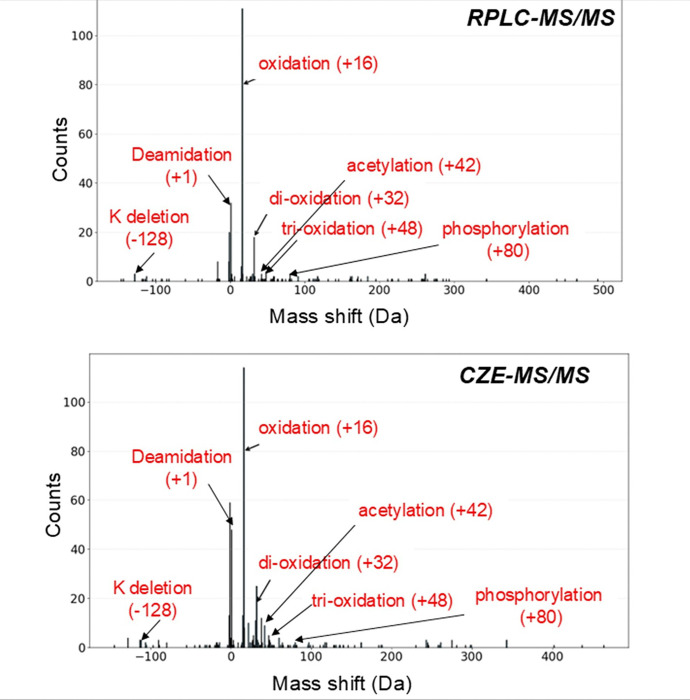
Distributions of matched mass shifts between BUP and TDP data using the PTM-TBA software. Combinatory BUP and TDP data for the RPLC-MS/MS-based TDP dataset (top) and the CZE-MS/MS-based TDP dataset (bottom). The MSFragger BUP data was used here. Some common matched PTMs were labelled on the figures.

Utilizing this integrated pipeline, we successfully matched the BUP PTM/mass-shift data (MSFragger) with the TDP mass-shift data for hundreds of proteoforms—471 proteoforms from the CZE-MS/MS dataset (representing 35.9% of the 1,312 proteoforms containing mass shifts) and 331 proteoforms from the RPLC-MS/MS dataset (34.5% of the 958 proteoforms with mass shifts), Fig. 4.

The matched proteoform and peptide information are listed in the **Supporting Information**. The BUP and TDP combination approach allows us to confirm or determine some common PTMs on proteoforms, *e.g.*, oxidation, multiple oxidation combinations, deamidation, acetylation, phosphorylation, and lysine (K) deletion, Fig. 4. Many mass shifts in the identified proteoforms cannot be matched with the BUP data regarding PTMs because those mass shifts could be due to the combinations of different PTMs, and the current version of PTM-TBA software cannot handle this situation, which will be one focus of the future development of the software.

**Figure 5:**
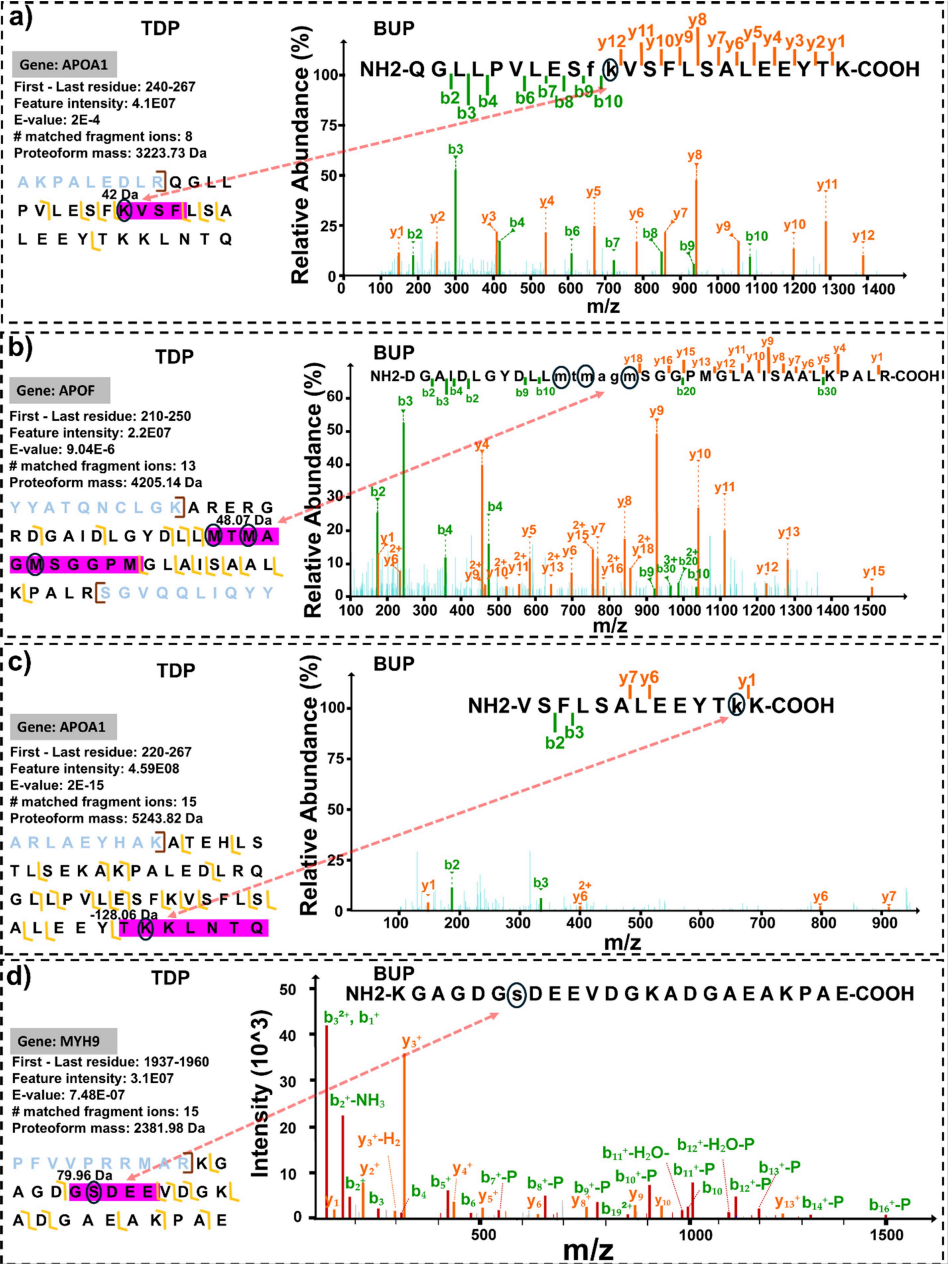
Representative examples of improved proteoform characterization using the combination of BUP and TDP data with the PTM-TBA pipeline. Four distinct cases are shown in which the PTM-TBA pipeline enabled confident identification and localization of PTMs or sequence variations on intact proteoforms. (A) one acetylation example; (B) one oxidation example; (C) Lysing deletion example; (D) one example of phosphorylation.

Figure 5 shows four examples of enhancing proteoform characterization quality by the combination of BUP and TDP. We observed a proteoform derived from myosin-9 (MYH9) carrying a + 79.96 Da mass shift. The integrated TDP–BUP analysis identified this modification as serine phosphorylation, supported by matching phosphopeptides detected in the bottom-up dataset (Fig. 5d). In another case, a prominent corona protein, —the major protein component of high-density lipoprotein (HDL) known for its protective roles against cardiovascular disease—exhibited a − 128.06 Da mass loss. Bottom-up sequencing revealed this to be a lysine deletion (Fig. 5c). Furthermore, an additional APOA1 proteoform displayed a + 42 Da mass shift. Without the combined analysis, this subtle PTM could have remained ambiguous; the bottom-up data confirmed it as lysine acetylation (Fig. 5a). Lysine acetylation is a well-established regulatory PTM that modulates protein function, interactions, and localization, underscoring the functional relevance of this modification in the protein corona environment. Finally, TDP revealed a proteoform from apolipoprotein F (APOF) with a + 48.07 Da mass shift, which, in conjunction with BUP data, was characterized as triple oxidation (Fig. 5b). **Figures S2-S4** illustrate additional examples, showing the improved determination and localization of modifications on proteoforms of Transthyretin (TTR) and apolipoprotein A-I (APOA1).

We further studied the proteoform profile differences of protein corona of human plasma samples from healthy controls and breast cancer patients (Grades I and II). Label-free quantification enabled measurement of proteoform abundances across groups (healthy vs. Grade I vs. Grade II). Differential expression analysis revealed differentially expressed proteoforms associated with disease progression: 115 proteoforms (from 23 genes) in the RPLC–MS/MS dataset (**Fig. S5**) and 31 proteoforms (from 10 genes) in the CZE–MS/MS dataset (**Fig. S6**). Those groups of differentially expressed proteoforms clearly separate the various disease conditions, documenting the potential of TDP-based protein corona analysis for disease diagnosis. The combination of TDP and BUP also improved the characterization of the differentially expressed proteoforms, **Fig. S5**. A notable case was an apolipoprotein C-II (APOC2) proteoform, markedly enriched in Grade II samples compared to Grade I and healthy controls. Top-down analysis showed a + 16 Da mass shift, consistent with single oxygen addition, and bottom-up sequencing confirmed methionine oxidation (methionine sulfoxide) at a defined site. Methionine oxidation is a hallmark of oxidative stress^51^, and the enrichment of this oxidized APOC2 proteoform in Grade II patients likely reflects the elevated oxidative environment of advanced cancer, with possible implications for APOC2’s role in lipid metabolism and corona interactions. Another example involved an apolipoprotein B-100 (APOB) proteoform, abundant in healthy samples but depleted in both patient groups. This proteoform carried a + 31.98 Da shift, identified as dihydroxylation, which was localized to a specific APOB region by bottom-up analysis. The loss of this modified APOB proteoform in cancer patients underscores how PTM-defined proteoforms can distinguish health from disease within the plasma corona.

Overall, our results demonstrate that the integration of TDP and BUP strategies significantly enhances our ability to accurately characterize proteoforms and their PTMs within complex protein corona. This comprehensive approach will advance the field of nanomedicine by providing an accurate proteome landscape in protein corona and offering critical insights into how specific PTMs may influence protein behavior, surface affinity, and nanoparticle interactions, thereby advancing our understanding of proteoform diversity in disease contexts.

## Conclusions

This study pioneers the integration of BUP and TDP data for the accurate characterization of proteoform landscape in protein corona. The novel approach markedly advances the precise characterization of proteoforms and their PTMs (i.e., types and localizations) within the protein corona. By combining the strengths of both approaches—TDP providing intact proteoform information and bottom-up offering detailed PTM localization—we achieve a level of resolution and confidence unattainable by either method alone. The development of the PTM-TBA pipeline further enhances data integration, enabling accurate PTM annotation and site-specific localization across complex biological samples. Our findings highlight the critical influence of PTMs on protein–nanoparticle interactions and highlight the importance of proteoform-level analysis in nanomedicine research. This comprehensive methodology also enables precise localization of modifications and revealing proteoform diversity associated with disease states. The observed differences in PTM abundances across healthy and breast cancer samples demonstrate the potential of proteoform profiling in biomarker discovery and personalized nanomedicine applications. Ultimately, this comprehensive characterization approach offers valuable insights into nanoparticle biodistribution, biosystem interactions, and proteoform-based biomarker discovery, paving the way for improved design and application of nanomedicines with enhanced safety and efficacy.

## Supplementary Material

Supplementary Files

This is a list of supplementary files associated with this preprint. Click to download.

• SupportingInformation.docx

• SupplementaryMovie1.mov

• Supportingdata.xlsx

## Figures and Tables

**Figure 1:**
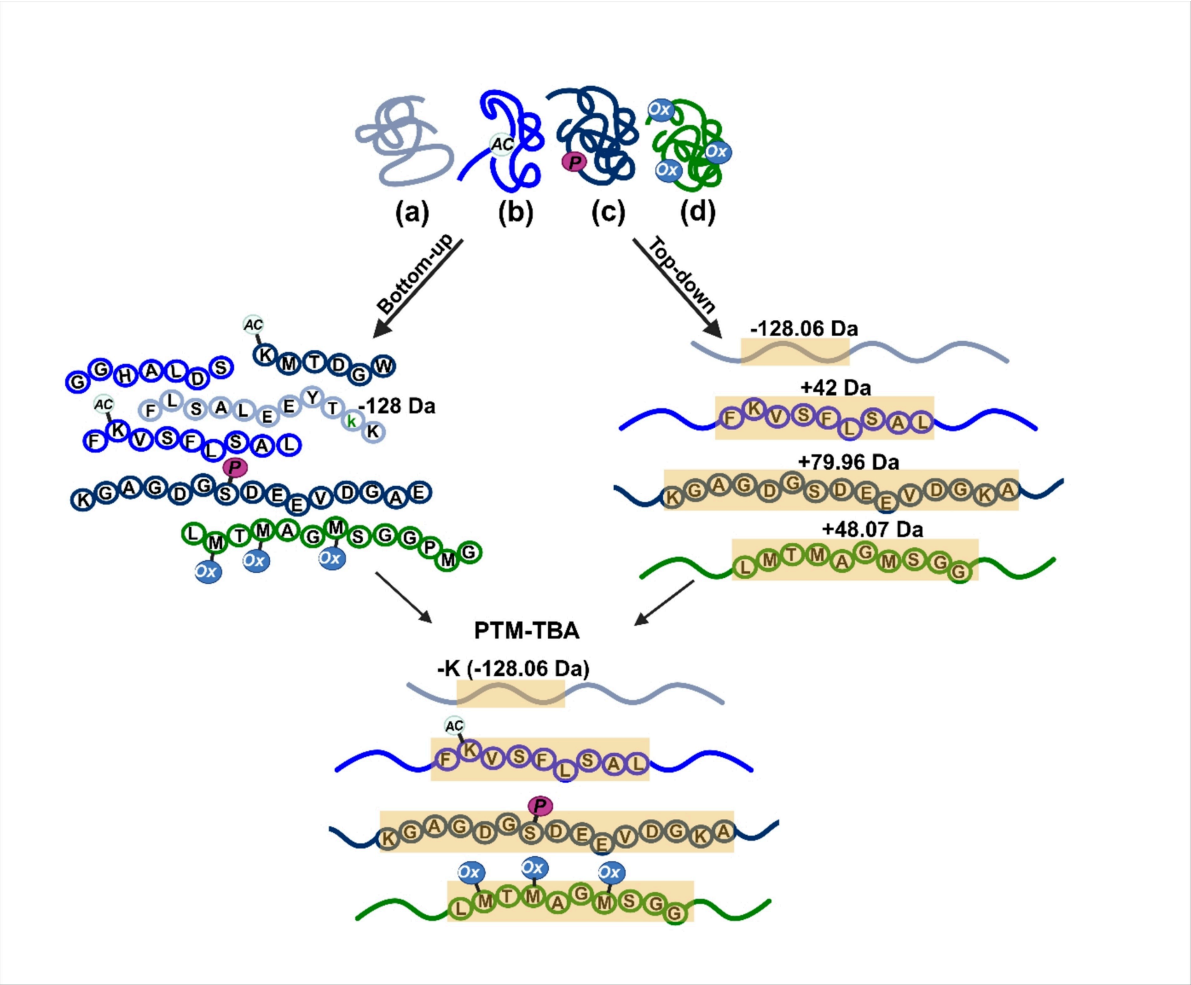
Schematic illustration of enhanced proteoform characterization achieved by integrating top-down and bottom-up proteomics using the PTM-TBA pipeline.
